# *Helicobacter pylori* infection eradication for nonalcoholic fatty liver disease: a randomized controlled trial

**DOI:** 10.1038/s41598-022-23746-0

**Published:** 2022-11-14

**Authors:** Ying-ying Yu, Yu-ling Tong, Ling-yan Wu, Xin-yan Yu

**Affiliations:** Department of General Practice, The Second Affiliated Hospital, Zhejiang University School of Medicine, No. 88 Jiefang Road, Hangzhou, 310009 Zhejiang China

**Keywords:** Helicobacter pylori, Non-alcoholic fatty liver disease

## Abstract

Previous studies have suggested that *Helicobacter pylori* (*H. pylori*) infection is associated with nonalcoholic fatty liver disease (NAFLD). The purpose of the present study was to investigate the effect of *H. pylori* eradication treatment on NAFLD patients. Two hundred NAFLD patients who tested positive for *H. pylori* infection were randomized into the *H. pylori* eradication treatment group or the control group. Metabolic and inflammatory parameters and FibroScan were measured in all subjects at baseline and 1 year after treatment. At 1 year after treatment, the decrease in metabolic indicators, such as fasting blood glucose, glycosylated haemoglobin, homeostasis model assessment of insulin resistance (HOMA-IR), triglycerides, body mass index and controlled attenuation parameter values, were more obvious in the treatment group. Moreover, the inflammatory indicators white blood count and high-sensitivity C-reactive protein (hs-CRP) and the inflammatory factors interleukin 6 (IL-6) and tumour necrosis factor-α (TNF-α) were also significantly decreased. *H. pylori* eradication can further reduce the metabolic indices of NAFLD and the degree of liver steatosis. *H. pylori* infection may participate in the occurrence and development of NAFLD through its influence on inflammatory factors. Thus, checking for the presence of *H. pylori* infection in patients at risk of NAFLD may be beneficial.

## Introduction

*Helicobacter pylori (H. pylori)* causes significant morbidity and mortality in gastric cancer, peptic ulcers, and chronic gastritis, which infect more than half of the world’s population^1^. The World Health Organization (WHO) has classified *H. pylori* as a class I carcinogen^2^.

Moreover, *H. pylori* infection has been defined as an infectious disease and is considered to be associated with many extragastrointestinal diseases^3,4^. Studies have suggested that *H. pylori* infection increases systemic inflammation by producing inflammatory factors, which results in the development of insulin resistance (IR) and metabolic syndrome (MetS)^5–7^. Therefore, *H. pylori* may be a risk factor for cardiovascular disease, diabetes and nonalcoholic fatty liver disease (NAFLD)^8–15^.

NAFLD is characterized by excessive fat deposition in liver cells, excluding secondary causes such as viral hepatitis, alcohol or hereditary liver diseases. It is closely related to IR and genetic susceptibility to metabolic stress-induced liver damage and ranges from isolated steatosis, nonalcoholic steatohepatitis (NASH) and cirrhosis^16,17^. Current routinely used modalities, such as laboratory tests and ultrasonography, cannot adequately determine the levels of steatosis and fibrosis, and liver biopsy is not accepted by most people. Among the noninvasive tests, transient elastography (FibroScan) has demonstrated good accuracy in quantifying the levels of liver steatosis and fibrosis in patients with NAFLD^18,19^.

Based on the above research, we hypothesized that *H. pylori* eradication treatment could reduce the inflammatory state of the body, thereby improving the pathophysiology of NAFLD. To our knowledge, few studies have evaluated the effects of *H. pylori* eradication treatment in NAFLD patients. Thus, we conducted a randomized controlled trial to investigate the effects of eradicating *H. pylori* infection in NAFLD patients.

## Results

### Clinical and demographic characteristics

**Figure 1:**
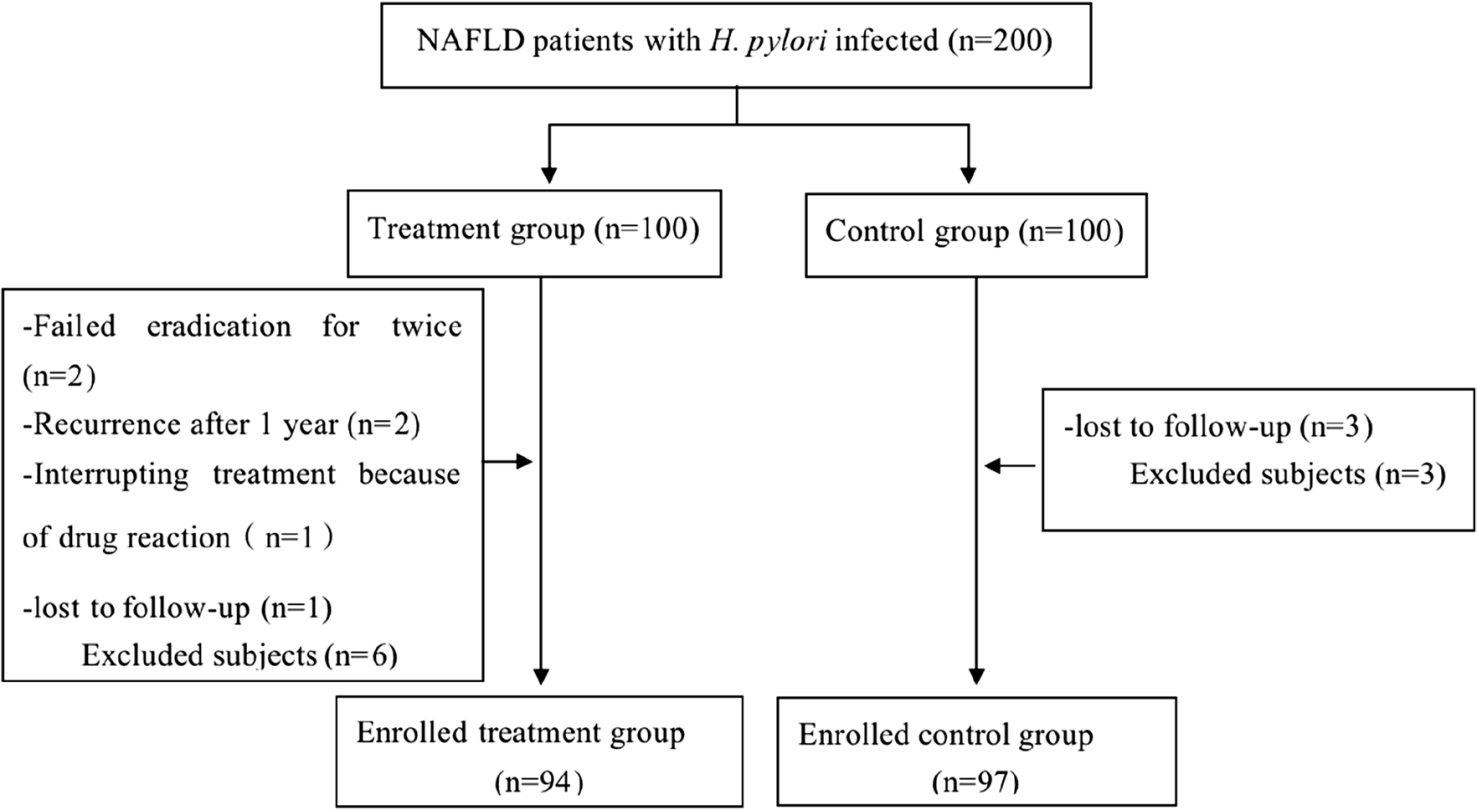
Flow diagram depicting the study.

In this study, a total of 200 NAFLD patients with positive *H. pylori* infection were randomly divided into two groups. In the treatment group of 100 cases, 6 cases failed the first eradication. After adjusting the treatment plan according to the guidelines, a second eradication treatment was performed. After the second eradication, 2 cases of eradication failure were referred to the gastroenterologist. One year after successful eradication treatment, 2 patients with positive 13-C UBT were re-examined and thus withdrew from the study. In addition, 4 cases were lost to follow-up during the study (1 in the treatment group and 3 in the control group), and 1 case did not formally complete the eradication treatment due to a drug reaction (Fig. 1). Finally, a total of 94 patients were included in the treatment group. In this group, the average age was 50.20 ± 12.13 years old, and 65.2% were males. The *H. pylori* infection eradication percentage was 94%. Ninety-seven patients in the control group had an average age of 50.01 ± 10.11 years old, among whom 69.1% were males.

### Comparison of the metabolic index, inflammatory parameters and FibroScan CAP values before and after treatment in the control group

In the control group, the diastolic blood pressure (80.85 ± 10.71 vs. 79.11 ± 8.61, *p* = 0.001), systolic blood pressure (133.7 ± 16.09 vs. 132.2 ± 13.44, *p* = 0.007), BMI (26.36 ± 2.09 vs. 26.16 ± 1.02, *p* < 0.001), ALT (32.15 ± 17.84 vs. 29.39 ± 13.26, *p* < 0.001), AST (26.81 ± 10.61 vs. 25.58 ± 9.88, *p* < 0.001), r-GT (36.53 ± 19.47 vs. 32.2 ± 15.30, *p* < 0.001), fasting blood glucose (5.55 ± 0.71 vs. 5.38 ± 0.63, *p* < 0.001), glycosylated haemoglobin (6.08 ± 0.76 vs. 5.80 ± 0.67, *p* < 0.001), HOMA-IR (3.11 ± 0.98vs 2.91 ± 0.97, *p* < 0.001), triglyceride (1.90 ± 0.94 vs. 1.67 ± 0.64, *p* < 0.001) and hepatic steatosis CAP values (245.53 ± 15.43 vs. 233.24 ± 10.29, *p* < 0.001) were significantly decreased. The inflammation indices WBC (6.83 ± 1.48 vs. 6.73 ± 1.26, *p* = 0.186) and hs-CRP (1.82 ± 1.99 vs. 1.66 ± 1.58, *p* = 0.088) and the inflammatory factors IL-6 6 (25.03 ± 4.09 vs. 24 ± 3.91, *p* = 0.063) and TNF-α (37.42 ± 6.71 vs. 35.68 ± 6.53, *p* = 0.088) were all decreased slightly, although significant differences were not observed (Table 1).

**Table 1:** Comparison of therapeutic effects before and after treatment in the control group.

Variable	Before treatment	After treatment	*t*	*p value*
DBP (mmHg)	80.85 ± 10.71	79.11 ± 8.61	3.518	0.001
SBP (mmHg)	133.7 ± 16.09	132.2 ± 13.44	2.758	0.007
BMI (kg/m^2^)	26.36 ± 2.09	26.16 ± 1.02	6.834	0.000
ALT (U/L)	32.15 ± 17.84	29.39 ± 13.26	4.058	0.000
r-GT (U/L)	36.53 ± 19.47	32.2 ± 15.30	6.452	0.000
AST (U/L)	26.81 ± 10.61	25.58 ± 9.88	4.255	0.000
FBG (mg/dL)	5.55 ± 0.71	5.38 ± 0.63	8.404	0.000
HbA1c (%)	6.08 ± 0.76	5.80 ± 0.67	12.806	0.000
HOMA-IR	3.11 ± 0.98	2.91 ± 0.97	7.378	0.000
TG (mmol/L)	1.90 ± 0.94	1.67 ± 0.64	3.947	0.000
CAP value (dB/m)	245.53 ± 15.43	233.24 ± 10.29	4.553	0.000
WBC (× 10^9^/L)	6.83 ± 1.48	6.73 ± 1.26	0.416	0.186
hs-CRP (mg/L)	1.82 ± 1.99	1.66 ± 1.58	1.186	0.088
IL-6 (pg/ml)	25.03 ± 4.09	24 ± 3.91	1.652	0.063
TNF-α (pg/ml)	37.42 ± 6.71	35.68 ± 6.53	1.290	0.070

*SBP* systolic blood pressure, *DBP* diastolic blood pressure, *BMI* body mass index, *WBC* white blood cell, *ALT* alanine aminotransferase, *γ-GT* γ-glutamyltranspeptidase, *AST* aspartate aminotransferase, *hs-CRP* high-sensitivity C-reactive protein, *FBG* fasting blood glucose, *HbA1c* glycosylated haemoglobin, *HOMA-IR* homeostasis model assessment of insulin resistance, *TG* triglyceride, *CAP* controlled attenuation parameter, *IL-6* interleukin 6, *TNF-α* tumour necrosis factor-α.

*p value obtained from paired t test.

### Comparison of the metabolic index, inflammatory parameters and FibroScan CAP values before and after treatment in the eradication group

In the *H. pylori* treatment group, the diastolic blood pressure (81.68 ± 11.27 vs. 78.34 ± 7.16, *p* < 0.001), systolic blood pressure (134.79 ± 16.67 vs. 131.28 ± 10.46, *p* < 0.001), BMI (25.99 ± 2.55 vs. 25.56 ± 2.19, *p* < 0.001), ALT (38.42 ± 22.32 vs. 33.89 ± 16.66, *p* < 0.001), AST (29.38 ± 12.18 vs. 25.64 ± 7.64, *p* < 0.001), r-GT (41.21 ± 21.38 vs. 34.05 ± 15.51, *p* < 0.001), fasting blood glucose (5.70 ± 0.91 vs. 5.32 ± 0.72, *p* < 0.001), glycosylated haemoglobin (5.87 ± 1.06 vs. 5.35 ± 0.81, *p* < 0.001), HOMA-IR (3.48 ± 1.40 vs. 3.07 ± 1.18, *p* < 0.001), triglyceride (2.07 ± 1.72 vs. 1.66 ± 0.67, *p* < 0.001) and hepatic steatosis CAP values (250.36 ± 17.15 vs. 226.34 ± 11.42, *p* < 0.001) were also significantly reduced after treatment. The inflammation indices WBC (7.06 ± 1.85 vs. 6.44 ± 1.45, *p* < 0.001) and hs-CRP (2.22 ± 3.33 vs. 1.46 ± 1.12, *p* < 0.001) and the inflammatory factors IL-6 (25.96 ± 5.91 vs. 21.10 ± 3.96, *p* < 0.001) and TNF-α (36.64 ± 7.23 vs. 28.55 ± 6.85, *p* < 0.001) were also significantly decreased (Table 2).

**Table 2:** Comparison of therapeutic effects before and after treatment in the treatment group.

Variable	Before treatment	After treatment	*t*	*p value*
DBP (mmHg)	81.68 ± 11.27	78.34 ± 7.16	4.729	0.000
SBP (mmHg)	134.79 ± 16.67	131.28 ± 10.46	3.527	0.000
BMI (kg/m^2^)	25.99 ± 2.55	25.56 ± 2.19	6.273	0.000
ALT (U/L)	38.42 ± 22.32	33.89 ± 16.66	4.929	0.000
r-GT (U/L)	41.21 ± 21.38	34.05 ± 15.51	5.624	0.000
AST (U/L)	29.38 ± 12.18	25.64 ± 7.64	4.646	0.000
FBG (mg/dL)	5.70 ± 0.91	5.32 ± 0.72	7.443	0.000
HbA1c (%)	5.87 ± 1.06	5.35 ± 0.81	8.274	0.000
HOMA-IR	3.48 ± 1.40	3.07 ± 1.18	5.864	0.000
TG (mmol/L)	2.07 ± 1.72	1.66 ± 0.67	5.027	0.000
CAP value (dB/m)	250.36 ± 17.15	226.34 ± 11.42	9.362	0.000
WBC (× 10^9^/L)	7.06 ± 1.85	6.44 ± 1.45	7.527	0.000
hs-CRP (mg/L)	2.22 ± 3.33	1.46 ± 1.12	2.705	0.008
IL-6 (pg/ml)	25.96 ± 5.91	21.10 ± 3.96	17.738	0.000
TNF-α (pg/ml)	36.64 ± 7.23	28.55 ± 6.85	18.213	0.000

*SBP* systolic blood pressure, *DBP* diastolic blood pressure, *BMI* body mass index, *WBC* white blood cell, *ALT* alanine aminotransferase, *γ-GT* γ-glutamyltranspeptidase, *AST* aspartate aminotransferase, *hs-CRP* high-sensitivity C-reactive protein, *FBG* fasting blood glucose, *HbA1c* glycosylated haemoglobin, *HOMA-IR* homeostasis model assessment of insulin resistance, *TG* triglyceride, *CAP* controlled attenuation parameter, *IL-6* interleukin 6, *TNF-α* tumour necrosis factor-α.

*p value obtained from paired t test.

### Comparison of the therapeutic effects between the treatment group and the control group

Before treatment, there was no statistically significant difference between the two groups in the metabolic indices at the basal value (*p* > 0.05). At 1 year after treatment, the treatment group presented a more obvious decrease in metabolic indicators, such as fasting blood glucose (0.38 ± 0.05 vs. 0.17 ± 0.02, *p* < 0.001), glycosylated haemoglobin (0.51 ± 0.06 vs. 0.28 ± 0.02, *p* = 0.001), HOMA-IR (0.41 ± 0.07 vs. 0.23 ± 0.03, *p* = 0.019), triglyceride (0.72 ± 0.14 vs. 0.20 ± 0.05, *p* = 0.001), body mass index (0.43 ± 0.07 vs. 0.20 ± 0.03, *p* = 0.003) and CAP values (24.02 ± 1.87 vs. 15.29 ± 0.84, *p* < 0.001), than the control group.

The inflammatory indicators WBC (24.02 ± 1.87 vs. 15.29 ± 0.84, *p* < 0.001) and hs-CRP (0.76 ± 0.28 vs. 0.16 ± 0.08, *p* = 0.045) and the inflammatory factors IL-6 (5.86 ± 0.33 vs. 1.03 ± 0.12), *p* < 0.001) and TNF-α (8.09 ± 0.44 vs. 1.74 ± 0.13, *p* < 0.001) were also significantly decreased in the treatment group.

**Figure 2:**
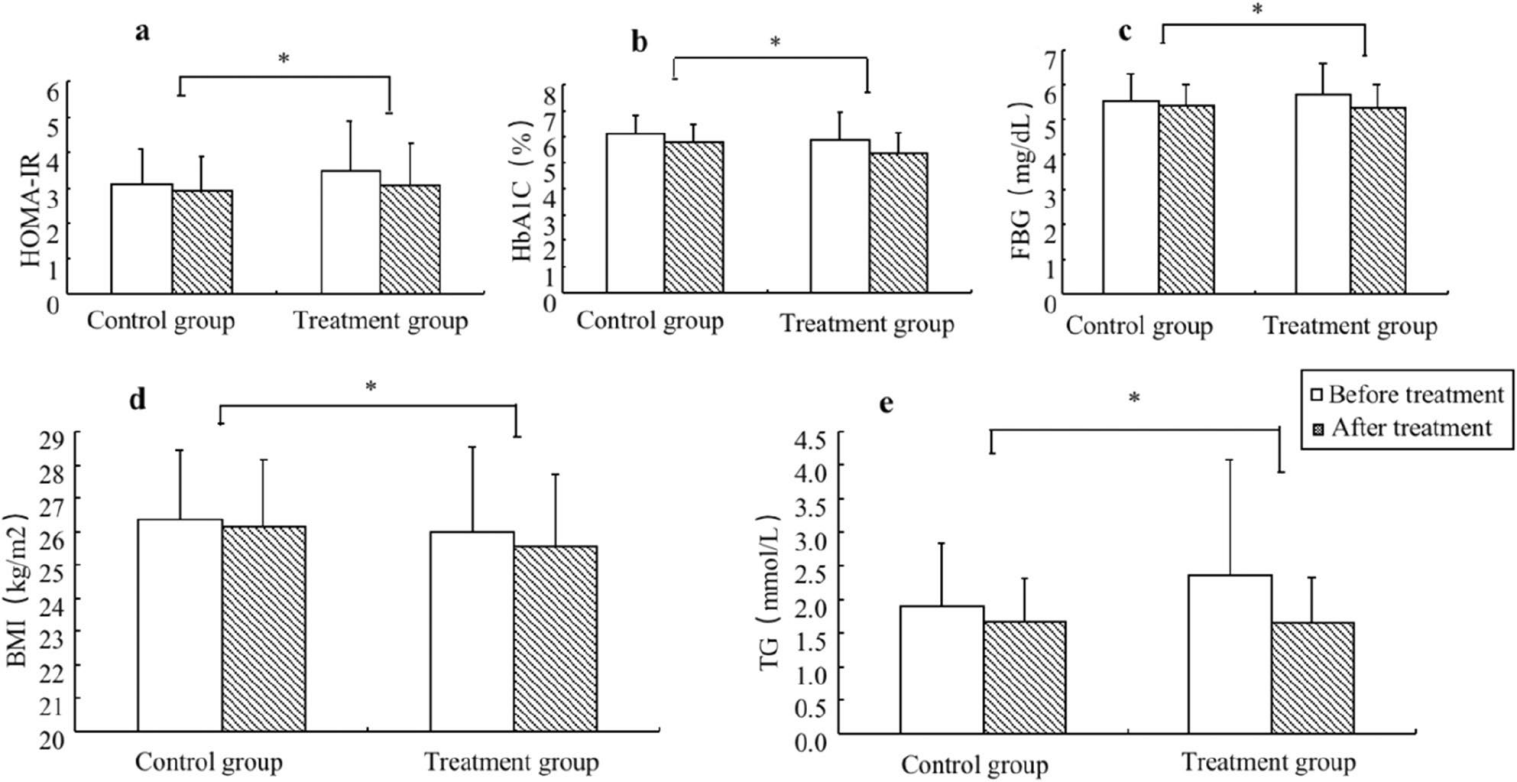
The treatment group presented a more robust decrease in metabolic indicators such as homeostasis model assessment of insulin resistance (**a**), glycosylated hemoglobin (**b**), fasting blood glucose (**c**), body mass index (**d**) and triglyceride (**e**) compared with the control group. * *p* < 0.05; HOMA-IR = homeostasis model assessment of insulin resistance, *HbA1c* glycosylated haemoglobin, *FBG* fasting blood glucose, *BMI* body mass index, *TG* triglyceride.

**Figure 3:**
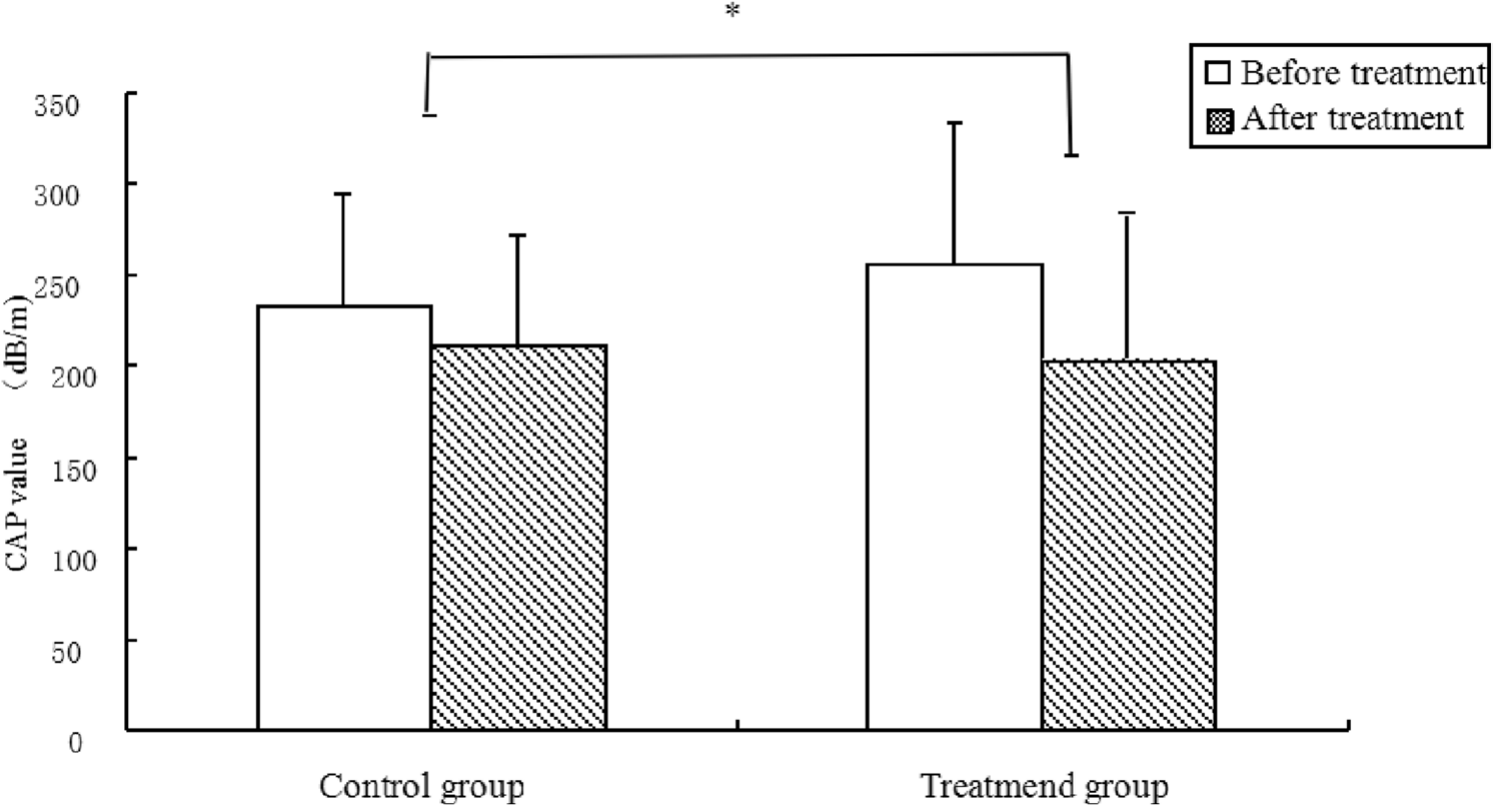
The treatment group presented a more robust decrease in CAP values than the control group. * *p* < 0.05; *CAP* controlled attenuation parameter.

**Figure 4:**
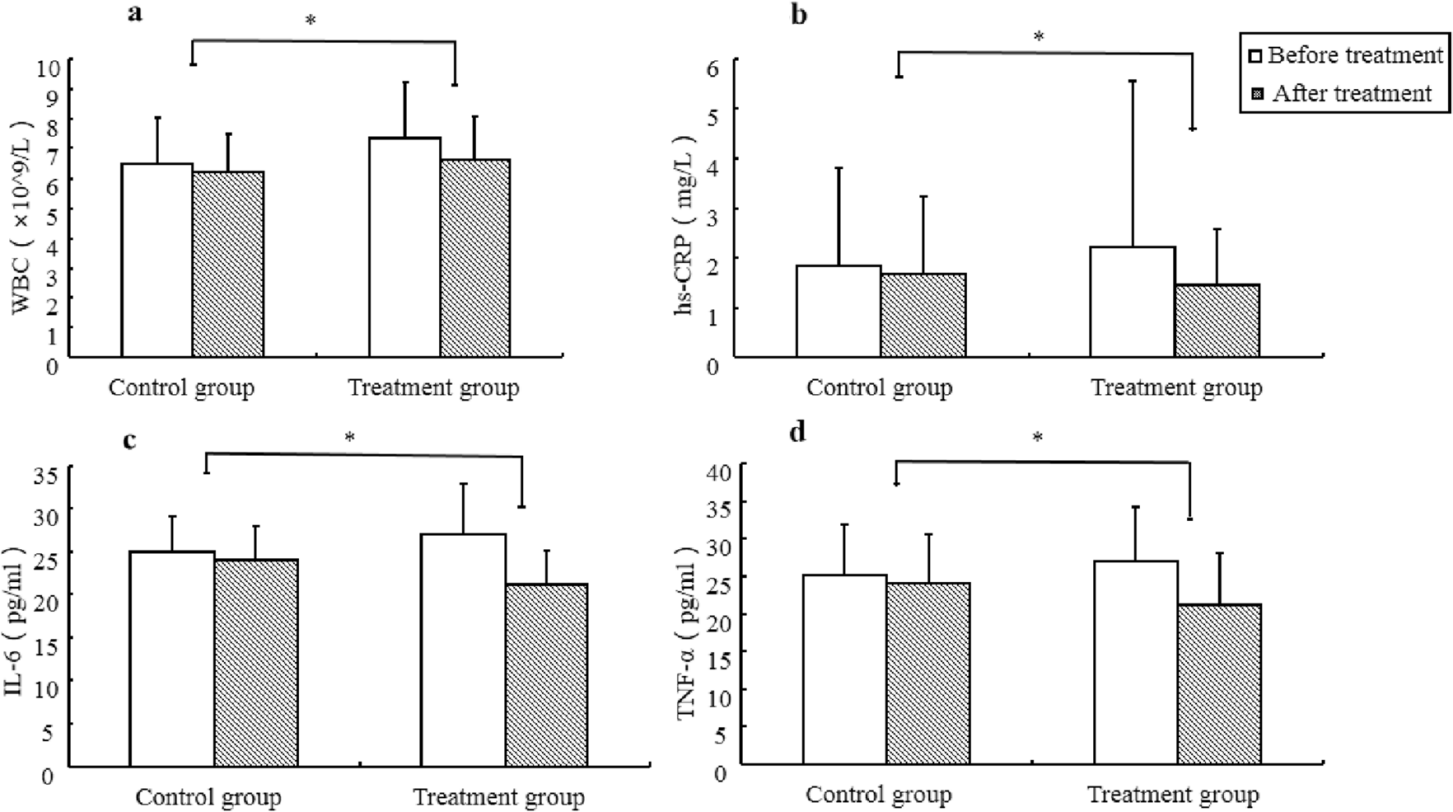
The treatment group presented a more robust decrease in inflammatory indicators white blood count (**a**) and high-sensitivity C-reactive protein (**b**) and the inflammatory factors Interleukin 6 (**c**) and Tumor necrosis facter-α (**d**) than the control group. * *p* < 0.05; *WBC* white blood cell, *hs-CRP* high-sensitivity C-reactive protein, *IL-6* interleukin 6, *TNF-α* tumor necrosis factor-α.

There was no significant difference between the two groups in the degree of decline of the liver function indices ALT (4.53 ± 0.92 vs. 2.76 ± 0.68, *p* = 0.125), AST (2.74 ± 0.80 vs. 1.24 ± 0.29, *p* = 0.051), and r-GT (7.16 ± 1.27 vs. 4.33 ± 0.67, *p* = 0.054) (Table 3 and Figs. 2, 3, 4).

**Table 3:** Comparison of therapeutic effects between the treatment group and the control group.

Variable	Time	Control group (*n* = 97)	Treatment group (*n* = 94)	***t***	*p value*
DBP (mmHg)	Before treatment	80.85 ± 10.71	81.68 ± 11.27	0.474	0.636
	After treatment	79.11 ± 8.61	78.34 ± 7.16	0.609	0.544
	d	1.74 ± 0.49	3.34 ± 0.71	1.861	0.065
SBP (mmHg)	Before treatment	133.7 ± 16.09	134.79 ± 16.67	1.110	0.269
	After treatment	132.2 ± 13.44	131.28 ± 10.46	1.000	0.319
	d	1.50 ± 0.54	0.51 ± 0.97	0.885	0.378
FBG (mg/dL)	Before treatment	5.55 ± 0.71	5.70 ± 0.91	1.123	0.263
	After treatment	5.38 ± 0.63	5.32 ± 0.72	0.540	0.590
	d	0.17 ± 0.02	0.38 ± 0.05	3.728	0.000*
HbA1c (%)	Before treatment	6.08 ± 0.76	5.87 ± 1.06	1.467	0.145
	After treatment	5.80 ± 0.67	5.35 ± 0.81	3.746	0.000
	d	0.28 ± 0.02	0.51 ± 0.06	3.504	0.001*
HOMA-IR	Before treatment	3.11 ± 0.98	3.48 ± 1.40	1.910	0.058
	After treatment	2.91 ± 0.97	3.07 ± 1.18	0.951	0.343
	d	0.23 ± 0.03	0.41 ± 0.07	2.390	0.019*
TG (mmol/L)	Before treatment	1.90 ± 0.94	2.07 ± 1.72	1.112	0.337
	After treatment	1.67 ± 0.64	1.66 ± 0.67	0.099	0.922
	d	0.20 ± 0.05	0.72 ± 0.14	3.406	0.001*
BMI (kg/m^2^)	Before treatment	26.36 ± 2.09	25.99 ± 2.55	0.994	0.322
	After treatment	26.16 ± 20	25.56 ± 2.19	1.785	0.076
	d	0.20 ± 0.03	0.43 ± 0.07	3.073	0.003*
ALT (U/L)	Before treatment	32.15 ± 17.84	38.42 ± 22.32	1.932	0.055
	After treatment	29.39 ± 13.26	33.89 ± 16.66	1.864	0.064
	d	2.76 ± 0.68	4.53 ± 0.92	1.543	0.125
r-GT (U/L)	Before treatment	36.53 ± 19.47	41.21 ± 21.38	1.433	0.154
	After treatment	32.2 ± 15.30	34.05 ± 15.51	0.751	0.454
	d	4.33 ± 0.67	7.16 ± 1.27	1.969	0.051
AST (U/L)	Before treatment	26.81 ± 10.61	29.38 ± 12.18	1.402	0.163
	After treatment	25.58 ± 9.88	26.64 ± 7.64	0.149	0.961
	d	1.24 ± 0.29	2.74 ± 0.80	1.983	0.054
CAP value (dB/m)	Before treatment	254.53 ± 15.43	260.36 ± 17.15	2.111	0.102
	After treatment	239.24 ± 10.29	236.34 ± 11.42	1.983	0.049
	d	15.29 ± 0.84	24.02 ± 1.87	6.530	0.000*
WBC (× 10^9^/L)	Before treatment	6.83 ± 1.48	7.06 ± 1.85	1.081	0.262
	After treatment	6.73 ± 1.26	6.54 ± 1.45	0.583	0.549
	d	0.10 ± 0.04	0.52 ± 0.08	3.240	0.014*
hs-CRP (mg/L)	Before treatment	1.82 ± 1.99	2.22 ± 3.33	0.907	0.366
	After treatment	1.66 ± 1.58	1.46 ± 1.12	0.901	0.369
	d	0.16 ± 0.08	0.76 ± 0.28	2.039	0.045*
IL-6 (pg/ml)	Before treatment	25.03 ± 4.09	25.96 ± 5.91	1.355	0.420
	After treatment	24 ± 3.91	21.10 ± 3.96	4.600	0.000
	d	1.03 ± 0.12	5.86 ± 0.33	13.737	0.000*
TNF-α (pg/ml)	Before treatment	37.42 ± 6.71	36.64 ± 7.23	0.696	0.487
	After treatment	35.68 ± 6.53	28.55 ± 6.85	6.651	0.000
	d	1.74 ± 0.13	8.09 ± 0.44	13.708	0.000*

*SBP* systolic blood pressure, *DBP* diastolic blood pressure, *BMI* body mass index, *WBC* white blood cell, *ALT* alanine aminotransferase, *γ-GT* γ-glutamyltranspeptidase, *AST* aspartate aminotransferase, *hs-CRP* high-sensitivity C-reactive protein, *FBG* fasting blood glucose, *HbA1c* glycosylated haemoglobin, *HOMA-IR* homeostasis model assessment of insulin resistance, *TG* triglyceride, *CAP* controlled attenuation parameter, *IL-6* interleukin 6, *TNF-α* tumour necrosis factor-α.

*p value obtained from the SNK test.

## Discussion

In our study, the influence of lifestyle intervention on NAFLD was considered in the design. In addition to the comparison before and after treatment in the treatment group, a control group with only lifestyle intervention was specifically added. In addition, the hepatic steatosis CAP value was used to evaluate the treatment effect, which made the results more objective. Our prospective study demonstrated the significant effect of eradicating *H. pylori* infection in NAFLD patients. Thus, *H. pylori* eradication therapy may improve the therapeutic effect of NAFLD by reducing inflammatory indicators in the body.

Although controversy remains, increasing evidence has revealed that there is an association between *H. pylori* infection and NAFLD. In particular, Doulberis et al. favoured an association between active *H. pylori* infection and NAFLD severity in morbidly obese patients subjected to bariatric surgery. The histological diagnostic “gold standard” for both main variables of interest (active *H. pylori* infection and NAFLD) was used. Specifically, the rates of NASH, as well as hepatic steatosis, inflammation, and fibrosis, were higher in *H. pylori-*positive patients than in *H. pylori-*negative patients^20^. In addition, the global incidence of NAFLD and *H. pylori* infection are high^21,22^, and both increase with age. The potential clinical significance of research into these diseases lies in the fact that if the two are related, then eradicating *H. pylori* infection may prevent NAFLD from further developing into advanced diseases, such as liver cirrhosis and even hepatic cancer.

Only a few studies have evaluated the therapeutic effect of eradicating *H. pylori* infection on NAFLD. Polyzos et al. conducted a 12-month prospective study^11^, in which NAFLD patients confirmed by liver biopsy were selected and divided into an *H. pylori*-positive group and an *H. pylori*-negative group. The *H. pylori*-positive group was treated with eradication therapy, and hepatic steatosis, NAFLD fibrosis score and HSENSI (homocysteine, serum glutamic oxaloacetic transaminase, erythrocyte sedimentation rate, nonalcoholic steatohepatitis index) were used to evaluate the efficacy of *H. pylori* eradication treatment on NAFLD. The eradication of *H. pylori* had no long-term effect on hepatic steatosis but improved the NAFLD fibrosis score and HSENSI index. However, another randomized open-label trial compared lifestyle modification alone and lifestyle modification combined with eradicating *H. pylori* eradication therapy in NAFLD patients^23^. After follow-up for 6 months, the liver function (AST, ALT and r-GT), liver fat content and HOMA-IR were improved in both groups. However, the values did not differ between the two groups, and only dyspeptic NAFLD patients were included in the study.

A recently published study further established the relationship between *H. pylori* infection and NAFLD and provided evidence for eradicating *H. pylori* in patients with NAFLD^24^. This is a multicentre cohort study of 369 adults without NAFLD at baseline who were followed up for 2 years. *H. pylori* infection was detected by stool antigen, and the patients were divided into positive and negative groups. During the follow-up period, the researchers observed a higher incidence of NAFLD in *H. pylori*-positive individuals and no NAFLD in the *H. pylori*-negative group. After 2 years of follow-up, the *H. pylori*-positive NAFLD patients were further treated with eradication therapy based on lifestyle modification. The eradication of *H. pylori* reduced HIS and NAFLD liver fat scores. During the 2-year follow-up, 23 new NAFLD patients were diagnosed, 18 (78.3%) NAFLD patients achieved remission after *H. pylori* eradication treatment, and only 5 remained unchanged (21.7%). More importantly, the HOMA-IR, adipokines, and inflammatory markers, which are considered to be key indicators of the occurrence and development of NAFLD, were also significantly improved.

Studies have revealed that due to low-grade inflammation in obese and type 2 diabetic patients, the tissue and serum IL-6 levels are abnormally increased, which promotes the occurrence and development of chronic inflammation^25^. IL-6 can inhibit insulin-mediated lipolysis in white adipose tissue, thereby increasing the delivery of FFAs to the liver and promoting the occurrence and development of NAFLD^26^. Studies have reported that the use of anti-IL-6 antibodies to treat obese mice can increase insulin sensitivity, indicating that IL-6 is involved in the pathogenesis of liver IR^27^. Similarly, elevated levels of TNF-α can disrupt insulin signalling through serine phosphorylation, thereby inducing insulin resistance in adipocytes and surrounding tissues and leading to the development of T2DM^28^.

WBC and hs-CRP are the most commonly used inflammatory indicators in clinical practice, and their detection is simple, easy and inexpensive. Many studies have reported that the WBC level is positively correlated with NAFLD^29–31^. In addition, other studies have revealed that hs-CRP is also a promising biomarker for screening cardiovascular metabolic diseases and NAFLD^32,33^. Moreover, a retrospective cohort study by Lee et al. found that even within the normal range^33^, a higher level of hs-CRP was still an independent risk factor for NAFLD.

The results of our study showed that after only one year of treatment with correct lifestyle changes, the control group presented decreases in various metabolic indices and hepatic steatosis CAP values, and the difference was significant. This indicates that simply relying on lifestyle intervention has a therapeutic effect on NAFLD. Similarly, our study indicated that 1 year after *H. pylori* eradication treatment with lifestyle intervention, the metabolic indices and hepatic CAP values were also significantly lower than those before treatment, and the difference was significant.

Then, we further analysed the difference in the final efficacy between the two groups. The results of our study indicated that compared with the control group, the treatment group had a more significant decrease in metabolic indices and hepatic steatosis CAP values. This demonstrated that on the basis of lifestyle intervention, the eradicating *H. pylori* treatment can significantly improve the therapeutic effect in NAFLD patients. Moreover, the inflammatory indicators WBC and hs-CRP and inflammatory factors IL-6 and TNF-α were all significantly decreased in the treatment group compared with the control group. Therefore, we believe that eradicating *H. pylori* infection helps to delay the occurrence and development of NAFLD by decreasing inflammatory factors. These results can be also reflected in the clinical routine inflammation indicators WBC and hs-CRP; therefore, the therapeutic effect of NAFLD can be followed-up by monitoring the changes in WBC and hs-CRP in the clinic.

This study has some limitations. First, we diagnosed NAFLD by ultrasonography, which was not as accurate as biopsy but was easily accepted by patients. We also used the hepatic steatosis parameter CAP value to evaluate the treatment effect. Second, patients with obvious abnormal liver function are not suitable for simultaneous *H. pylori* eradication therapy. The enrolled subjects were selected based on abnormal liver enzyme index values less than 2 times the UNL, and those with hypertension, diabetes, and hyperlipidaemia under treatment were excluded. Thus, selection bias may have occurred, which may explain the lack of significant differences in the changes in liver function indices between the two groups.

In conclusion, our study revealed that for patients subject to the same lifestyle management, treatment to eradicate *H. pylori* infection can further reduce the metabolic indices of NAFLD and the degree of liver steatosis. *H. pylori* infection may participate in the occurrence and development of NAFLD through its influence on inflammatory factors in the body. Clinically, the therapeutic effect of NAFLD can be followed-up by monitoring changes in WBCs and hs-CRP. We hope that additional well-designed large randomized controlled studies with longer follow-up durations will be performed to further explore the relationship between *H. pylori* infection and NAFLD patients.

## Methods

### Study design

The study design was a randomized controlled trial (Fig. 1). Briefly, the NAFLD subjects were defined as *H. pylori* positive or negative by ^13^C-UBT. The *H. pylori*-positive subjects were randomized into two different groups by a random number table: treatment or no treatment. Four weeks after treatment, the presence of *H. pylori* infection was assessed again in the treated subjects by ^13^C-UBT. Metabolic and inflammatory parameters and FibroScan were measured in all subjects at baseline and 1 year after the treatment.

### Study participants

Participants who voluntarily underwent a general health screening from January to June 2017 were recruited from the International Health Care Centre of the Second Affiliated Hospital of Zhejiang University School of Medicine. The inclusion criteria were as follows: (1) ultrasonography for NAFLD; (2) alanine aminotransferase (ALT) levels less than 2 times the UNL; and (3) no symptoms of gastrointestinal tract disease and negative blood pepsinogen detection. The exclusion criteria were as follows: (1) the consumption of 3 or more alcoholic drink units per week; (2) any chronic liver disease; (3) a history of gastric surgery; (4) the use of bismuth, antibiotics, proton pump inhibitors or H2 blockers within the prior 4 weeks; (5) a significant mental or neurological disorder; (6) a history of cancer; and (7) patients on steatogenic medications, such as methotrexate and corticosteroids. All subjects underwent a detailed physical examination, including ^13^C-UBT detection of *H. pylori* infection, ultrasonography and FibroScan for NAFLD.

### Ethics approval and consent to participate

All participants provided written informed consent before the examination. The present study was reviewed and approved by the Ethics Committee of the 2nd Affiliated Hospital, School of Medicine, Zhejiang University (2016–322). All methods were carried out in accordance with the principles expressed in the Declaration of Helsinki.

### Registration

The trial was registered in the Chinese Clinical Trials Registry, with the name of the registry being “*Helicobacter pylori* Infection Eradication on Nonalcoholic Fatty Liver Disease: A Randomized Controlled Trial”. The trial registration number was ChiCTR2200061243 (retrospectively registered on 17/06/2022).

### Questionnaires

The medical history of each participant was obtained using a questionnaire that included the history of the present illness, previous diagnoses of *H. pylori* infection, history of anti-*H. pylori* therapy, history of gastric surgery, history of significant mental or neurological disorders, history of cancer(s), use of bismuth, antibiotics, PPIs or H_2_ blockers within the previous 4 weeks, alcohol intake, and cigarette smoking.

### Diagnosis of *H. pylori* infection

After fasting for at least 2 h, all participants underwent ^13^C-UBT at our centre. After a baseline breath sample was collected, the participants ingested a ^13^C-urea reagent that was dissolved in water. The second breath sample was collected 30 min later and analysed. A delta over baseline (DOB) value ≥ 4.0 indicated a positive result for *H. pylori* infection.

### Definition of NAFLD

NAFLD was defined according to the guidelines published in 2012 by the American Association for the Study of Liver Diseases (AASLD), American College of Gastroenterology (ACG), and American Gastroenterological Association (AGA)^34^. In this study, the diagnosis of NAFLD required the following: (1) hepatic steatosis detected by ultrasonography; (2) no significant alcohol consumption (to strictly exclude the influence of alcohol, we chose individuals with alcohol consumption of less than 3 drink units per week); and (3) no coexisting causes of chronic liver disease, such as hepatitis C, medications, parenteral nutrition, Wilson’s disease or severe malnutrition.

### Treatments

The enrolled NAFLD subjects were randomized to *H. pylori* eradication treatment or untreated groups based on standard instructions for diet and exercise according to the guidelines. The *H. pylori* eradication treatment consisted of 14 days of quadruple therapy (Esomeprazole 20 mg given twice daily + colloidal bismuth pectin 200 mg twice daily + amoxicillin 1000 mg twice daily + furazolidone 100 mg twice daily). If the patient was allergic to amoxicillin, it was substituted with clarithromycin (500 mg twice daily). Eradication was assessed by ^13^C-UBT 4 weeks after the end of treatment. Eradication was considered successful when the UBT was negative.

The untreated groups only accepted health education and lifestyle guidance.

### Health education and lifestyle guidance

Two general practitioners (unknown about the research group) provided full-time guidance and monthly telephone or network follow-up supervision.

The specific content was based on the recommendations of the guidelines.

1. Moderate-intensity calorie restriction and personalized daily calorie intake according to body mass index were recommended.
2. Changes in the diet composition to a low-sugar and low-fat balanced diet. Reductions in sucrose beverages, saturated fats and trans fats and increases in fibre content were recommended.
3. A moderate amount of aerobic exercise was recommended 5 times a week, and the cumulative exercise time should be at least 150 min.

### Data collection

Blood pressure measurements were obtained after at least 10 min of rest. Body mass index (BMI) was defined as weight divided by height squared (kg/m^2^). The waist circumference (WC) was measured while standing with a measuring tape midway between the lowest rib and the iliac crest. The fasting plasma WBC, hs-CRP, fasting blood glucose, glycosylated haemoglobin (HbA1c), alanine aminotransferase (ALT), γ-glutamyltranspeptidase (γ-GT), aspartate aminotransferase (AST), total cholesterol (TC), high-density lipoprotein (HDL-C), low-density lipoprotein (LDL-C), and triglyceride (TG) levels were measured after an 8-h overnight fast (Beckman Coulter AU 5400). HOMA-IR was calculated according to the following formula: HOMA-IR = [FINS (µIU/mL) × FPG (mmol/L)]/22.5.

### IL-6 and TNF-α

Venous blood (4 ml) was collected into a serum separation tube. After the blood clot formed, the sample was centrifuged, and the serum was collected. The total IL-6 and TNF-α contents in plasma were determined using ELISA (Abcam, USA).

### FibroScan

Liver stiffness was evaluated using a FibroScan-502 touch (Echosense, Paris, France). The operation was carried out according to the user manual by two specially trained doctors who obtained the FibroScan operator certificate.

1. Fasting patients were in the supine position, and they held their head with their right hand to maximize the expansion of the intercostal space.
2. The probe was placed on the right lobe of the liver in the intercostal position.
3. The probe was kept perpendicular to the skin. When the pressure indicator was displayed in green, the M waveform on the display screen was consistent and evenly distributed, and the A waveform was linear, subsequently, the detection process started.
4. The unit of liver steatosis was dB/m, which was achieved in 10 replications with a success rate of 60% and an interquartile range of < 30%.

### Statistical analysis

The statistical analysis was performed using SPSS 13.0 software. Measurement data are expressed as the mean ± standard deviation. A paired t test was used for comparisons before and after treatment within groups. A single factor analysis of variance was used for multigroup comparisons, and SNK was used for pairwise comparisons between groups. All *p* values were based on a two-sided test of statistical significance. Significance was accepted at the level of *p* < 0.05.

## Data Availability

The full trial protocol can be found at the Chinese Clinical Trials Registry (http://www.medresman.org.cn/uc/projectsh/projectedit.aspx?proj=4531). The datasets used and/or analysed during the current study are available from the corresponding author on reasonable request.
